# Valoración de los parámetros de investigación de los analizadores hematológicos de la serie XN (Sysmex^®^) como marcadores de displasia en sangre perifèrica

**DOI:** 10.1515/almed-2024-0111

**Published:** 2024-12-02

**Authors:** Vicente Aguadero, María López-Molina, Míriam Ruíz, Diana Regidor, Gemma Celma

**Affiliations:** Consorci del Laboratori Intercomarcal de l’Alt Penedès, l’Anoia i el Garraf (*CLILAB Diagnòstics*), Laboratori de l’Hospital Sant Joan Despí Moisès Broggi, Sant Joan Despí, Barcelona, España; Consorci del Laboratori Intercomarcal de l’Alt Penedès, l’Anoia i el Garraf (*CLILAB Diagnòstics*), Laboratori de Vilafranca del Penedès, Barcelona, España

**Keywords:** displasia, granulación de neutròfilos (Neu-Gi), ancho de distribución plaquetar (PDW), fracción de plaquetas inmaduras (IPF), Sysmex

## Abstract

**Objectivos:**

Los síndromes mielodisplásicos (SMD) son trastornos clonales hematopoyéticos caracterizados por citopenias en sangre periférica, displasia celular y riesgo de progresión a leucemia aguda. Estudios recientes reportan que algunos parámetros de investigación de los analizadores Sysmex XN-1000^®^, tales como fracción de plaquetas inmaduras (IPF), Índice de granulación de neutròfilos (Neu-GI), o ancho de distribución plaquetar (PDW), muestran relación con el hallazgo de displasia en sangre periférica. El objetivo es evaluar el grado de asociación de parámetros hematimétricos clásicos y de investigación con la presencia de displasia, y obtener un modelo multivariante que permita su predicción con elevada probabilidad.

**Métodos:**

Se estudiaron 75 pacientes mayores de 60 años con anemia, leucopenia o trombopenia, sin déficit de factores madurativos ni enfermedades hematológicas, utilizando el analizador XN-1000 (Sysmex).

**Resultados:**

Se observó displasia en el 32 % de los pacientes. Neu-GI, PDW e IPF muestran diferencias significativas entre pacientes con y sin displasia. Neu-GI reporta la mayor capacidad predictiva (AUC=0,98), sin que la adición de PDW o IPF la incremente de forma significativa. Un valor de Neu-GI≤146ch predice displasia con VPP=90 %.

**Conclusiones:**

Neu-GI es el parámetro más asociado con displasia. Un valor Neu-GI ≤146ch indica alta probabilidad de displasia y justifica la revisión del frotis sanguíneo, mientras que valores >152ch sugieren baja probabilidad de displasia.

## Introducción

Los síndromes mielodisplásicos (SMD) constituyen un grupo heterogéneo de alteraciones clonales de la hematopoyesis, caracterizados por presentar, distintos grados de citopenias en sangre periférica, alteraciones morfológicas características en los elementos hematopoyéticos (cambios displásicos), y un riesgo incrementado de progresión hacia leucemia aguda mieloblástica [1], [2], [3].

Se debe descartar la existencia de SMD cuando un paciente presenta anemia normo-macrocítica sin déficit de factores madurativos ni alteración hepática, que además curse con leucopenia o trombopenia [4].

El primer paso diagnóstico consiste en un estudio de hematimetría básica y la observación al microscopio de la extensión de sangre periférica, para la detección de posibles cambios displásicos [4]. En este paso, además de los clásicos parámetros de la hematimetría, los autoanalizadores hematológicos de la Serie XN de Sysmex, proporcionan una serie de parámetros, algunos de ellos todavía en investigación, que pudieran relacionarse con la presencia de displasia en sangre periférica [5]. Así, algunos estudios informan que parámetros como índice de granulación de neutrófilos (Neu-GI) o plaquetas inmaduras (IPF), muestran relación con la presencia de displasia, pudiendo ser potenciales alertas para el diagnóstico temprano de un SMD [6], 7]. Estos estudios, son todavía escasos y además valoran los parámetros de forma individualizada. Creemos que a partir de un estudio que englobe todos los parámetros hematimétricos, clásicos y novedosos, podríamos obtener una combinación de ellos, que mejore valores predictivos respecto a la presencia de displasia en sangre periférica.

### Objetivos

Determinar el grado de asociación de los parámetros clásicos y de investigación de Sysmex XN, por separado y en conjunto, con la presencia de displasia.

Obtener una fórmula, índice, o valor/es de corte, que nos permita predecir o descartar con elevada probabilidad la existencia de displasia, y por tanto, indicar si la revisión del frotis sanguíneo es imperiosa o prescindible.

## Materiales y métodos

Durante 6 meses se incorporan al estudio pacientes que en un análisis rutinario del hemograma, realizado mediante el analizador hematológico XN-1000 (Sysmex Corporation, Kobe, Japan), cumplen los siguientes tres requisitos: a) edad superior a 60 años, b) anemia normocítica o macrocítica + leucopenia ó trombopenia, c) sin déficit de factores madurativos, hepatopatía, tratamiento oncológico, o histórico previo de enfermedad hematológica. Para cada uno se recoge la edad, el sexo, tipo de citopenia (anemia + trombopenia / anemia + leucopenia / pancitopenia), tipo de anemia (normocítica / macrocítica), los resultados de los parámetros clásicos del hemograma (hemoglobina, recuento leucocitario, recuento eritrocitario, recuento de plaquetas, ratio de los porcentajes de neutrófilos/monocitos, volumen corpuscular medio (VCM), y ancho de distribución leucocitario), de los parámetros de investigación (IPF, Neu-GI, ancho de distribución plaquetar (PDW), porcentaje de macrocitos (Macro-R), porcentaje de microcitos (Micro-R), porcentaje de reticulocitos inmaduros (IRF)), y el hallazgo de displasia (SI/NO) en cualquiera de las tres series celulares de la extensión de sangre periférica. Se consideran criterios de displasia [4], 8]:

– Diseritropoiesis: eritroblastos con disociación madurativa y binuclealidad; eritrocitos con inclusiones eritrocitarias (anillos de Cabot, cuerpos de Howell-Jolly y punteado basófilo).
– Disgranulopoiesis: hipogranulación, hipersegmentación, hiposegmentación (pseudo-pelguer), segmentación aberrante, mielocatexis, e inclusiones de cuerpo de Döhle.
– Dismegacariopoiesis: plaquetas de tamaño gigante, degranuladas, distribución anómala de la granulación, o presencia de pseudópodos.

La revisión del frotis sanguíneo es realizada por dos observadores experimentados. Se utiliza el test Chi-cuadrado de Pearson (χ^2^) para demostrar la ausencia de diferencias significativas en la valoración. Los pacientes son clasificados en dos grupos según la evaluación microscópica del frotis sanguíneo: hallazgo de displasia (DS) / ausencia de displasia (DN). La normalidad de las variables cuantitativas es evaluada mediante el test de Kolmogorov-Smirnoff. Se utilizan los test de t-Student ó U-Mann–Whitney para comparar las medias entre los grupos DS / DN de las variables cuantitativas, y χ^2^ para comparar los porcentajes de las cualitativas. Aquellas para las que obtenemos diferencias significativas, se introducen en un estudio de regresión logistica multivariante. Mediante el método “hacia atrás” (backward) se descartan las variables prescindibles para el algoritmo, escogiendo las variables con mayor poder predictivo de displasia, representado por el valor del estadístico de wald (EW). Los valores atípicos son detectados mediante la evaluación de las distancias de Cook. La fiabilidad de los modelos de regresión es evaluada mediante el área bajo la curva (AUC) y el porcentaje de exactitud (E). Se utiliza el test de Delong para la comparación entre las AUC de diferentes modelos de regresión. Los valores de corte se obtienen a partir de los puntos de coordenadas de las curvas ROC, que reportan los porcentajes de sensibilidad y/o especificidad deseados. Todos los análisis son realizados con los programas R Commander 3.6.1 y SPSS versión 25.0.

## Resultados

Se incorporan al estudio 75 pacientes. Se describe displasia en 24 casos (32 %). La media o el número de casos correspondiente a los grupos DS y DN para cada variable, se muestran en la Tabla 1.

**Tabla 1:** Recuento de casos y medias de cada uno de los parámetros hematimétricos estudiados en función del hallazgo o no de displasia en el frotis de sangre periférica.

	Displasia		Total	Sig
	No (casos/media)	Si (casos/media)		
Sexo
Hombre	34	16	50	0,361^a^
Mujer	18	7	25	
Total	49	21	75	
Citopenia
Anemia+leucopenia	2	1	3	0,337^a^
Anemia+trombopenia	21	14	35	
Pancitopenia	28	9	37	
Total	51	24	75	
Anemia
Normocítica	17	10	27	0,517^a^
Macrocítica	34	14	48	
Total	51	24	75	
Edad, años	76	78		0,103^b^
Hemoglobina, g/L	96,1	96,2		0,987^c^
Volumen corpuscular medio, FL	100,0	97,4		0,139^b^
Eritrocitos, 10^12^/L	2,90	3,01		0,214^b^
Leucocitos, 10^12^/L	4,75	7,72		0,120^c^
Plaquetas, 10^9^/L	65	77		0,286^c^
Ancho distribución eritrocitaria, %	16,2	16,1		0,791^c^
Neutrófilos, %	61,5	61,1		0,459^b^
Monocitos, %	11,1	10,9		0,987^c^
Ancho distribución plaquetar, FL	13,4	16,3		<0,001^b^
Plaquetas inmaduras, %	6,4	11,9		<0,001^c^
Neu-GI (SI)	153	144		<0,001^b^
Macrocitos, %	10,7	8,1		0,299^c^
Microcitos, %	2,1	2,4		0,134^c^
Reticulocitos inmaduros, %	20,0	20,7		0,378^b^
Neutrófilos, % / monocitos, %	6,94	7,8		0,728^c^

Sig, nivel de significación de la diferencia (se considera significativa con p<0.05). ^a^Test Chi-cuadrado; ^b^test T-Student; ^c^test U-Mann Whitney.

Los observadores no muestran diferencias significativas en la evaluación de los frotis sanguíneos (p<0.05, χ^2^).

**Figura 1:**
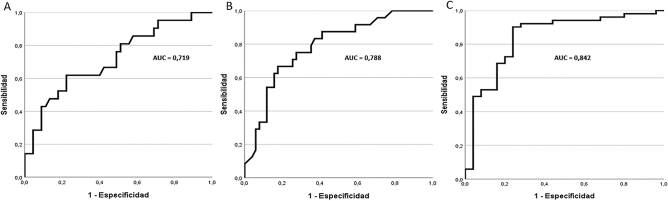
Curvas ROC de los parámetros que en el análisis estadístico bivariante muestran una asociación significativa con la presencia de cambios displásicos en el frotis de sangre periférica. (A) Ancho de distribución plaquetar (PDW), (B) porcentaje de plaquetas inmaduras (IPF), (C) indice de granulación de neutrófilos (Neu-GI).

El estudio bivariante reporta que la presencia de displasia no se asocia significativamente a ningún tipo de anemia o citopenia concreta. De los parámetros cuantitativos estudiados, solo las medias de Neu-GI, PDW y IPF presentan diferencias significatives entre los grupos DS y DN (Tabla 1). La presencia de displasia se relaciona con valores bajos de Neu-GI y valores altos de PDW e IPF, con valores de AUC de 0,842, 0,788 y 0,842 respectivamente (Figura 1).

**Figura 2:**
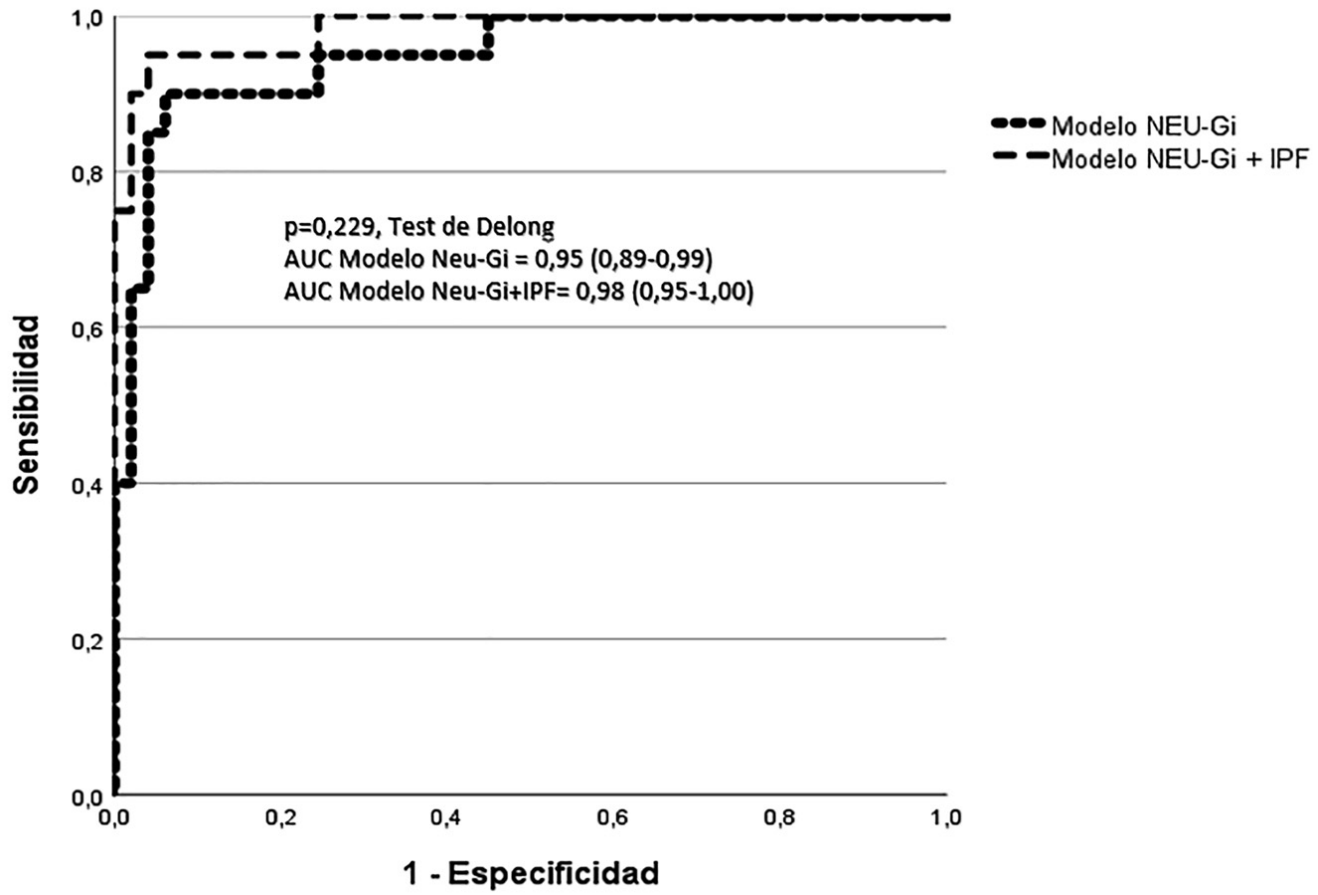
Comparación de las curvas ROC del modelo predictivo de displasia constituido por Neu-GI y IPF, y el constituido exclusivamente por Neu-GI. Diferencias significativas con p<0.05 (test de Delong).

En el análisis multivariante, PDW no muestra asociación significativa (p=0,47, EW=0,5). Neu-GI muestra el mayor poder de asociación (p=0,001, EW=10,2), seguido de IPF (p=0,030, EW=4,7). Se construyen dos modelos, uno constituido por Neu-GI+IPF (AUC=0.95, E=94 %), y otro solo por Neu-GI (AUC=0,98, E=92 %), que no muestran diferencias significativas en el poder predictivo de displasia (p=0,229, test de Delong) (Figura 2). Escogiendo como referente el modelo constituido únicamente por Neu-GI, y un valor de corte de ≤146 SI, se consigue predecir la presencia de displasia con un valor predictivo positivo del 90 % (83 % de sensibilidad, 96 % de especificidad, y 92 % valor predictivo negativo (VPN)). Para alcanzar VPN del 100 % se debe aumentar el valor de corte hasta 152 SI.

## Discusión

El valor de Neu-GI es obtenido a partir del índice de dispersión lateral del rayo láser que el citómetro de flujo fluorescente hace incidir en la célula y que es captado por el canal SSC (*side-scattered light*). Éste proporciona información sobre la estructura interna de la célula. Si la complejidad de esta estructura aumenta a raíz de un cambio en la funcionalidad (p. ej. aumento reactivo de la granulación tóxica en un estado inflamatorio, o disminución de ésta ante un defecto de formación de origen central), la posición del cúmulo de neutrófilos a lo largo del eje x en el diagrama de dispersión, se verá alterada, y el valor de Neu-GI expresado en la unidad SI (intensidad de dispersión) variará en consecuencia [9].

Nuestros resultados revelan que Neu-GI, es la variable que presenta una mayor asociación con displasia. Ésta es técnicamente, una evolución de los parámetros de investigación Neu-X y Neu-Y disponibles en los autoanalizadores de la serie XE de Sysmex, cuyo potencial como marcadores de displasia ya fue valorado positivamente, reportando competencia para detectar el 75 % de los casos de SMD que cursan con neutrófilos hipogranulados [10]. A pesar de ser un parámetro referenciado a la serie blanca, solo el 56 % de los casos de nuestro estudio presentan leucopenia. Esto otorga al parámetro más valor si cabe, pues se revela como un marcador de displasia, aún sin restricción cuantitativa de la serie blanca.

A pesar de que el tipo de anemia predominante es la macrocítica, ni el % de Macro-R ni el VCM muestran correlación con la presencia de displasia. Cierto es que existen estudios que reportan la asociación de VCM con el diagnóstico final de SMD, aunque no directamente con el hallazgo de cambios displásicos en sangre perfiférica [11], 12].

Es importante destacar que el 96 % de los casos presentan trombopenia, lo que podría haber potenciado la asociación de los parámetros relacionados con esta serie, como PDW y IPF. Así, la AUC que reportamos con respecto al hallazgo de displasia es superior a la reportada por otros estudios con respecto al dignóstico de SMD [13], 14].

Sorprende que siendo el SMD un trastorno medular de tipo central, valores bajos de IRF no se asocien con displasia. Esto si ocurre sin embargo con IPF, pero al contrario de lo que cabría pensar, y coincidiendo con lo ya reportado por otros grupos, se asocia con valores elevados. Esta circunstancia se explicaría porque en el ámbito de los pacientes con SMD, tasas porcentuales aberrantemente altas de IPF, reflejarían la presencia de distrombopoyesis en contraposición a un aumento de la actividad megacariocítica [15].

En este estudio se han escogido pacientes en los que en la práctica habitual, se sospecharía la existencia de un SMD, reforzado por la existencia, no solo de anemia, sino de otra citopenia añadida. Sin embargo, en una elevada proporción de pacientes, la única alteración hematimétrica en el momento del diagnóstico es la anemia, casos que no se encuentran representados en nuestro estudio. Quizá contando con esta casuística, los parámetros relacionados con la serie roja, como macro/micro-R, IRF, VCM o ADW, pudieran haber mostrado un mayor protagonismo en la predicción de displasia.

Los cambios displásicos no son patognomónicos de SMD, existen otras situaciones patológicas hematológicas (síndromes mieloproliferativos) y no hematológicas (déficits de factores madurativos, agentes tóxicos, neoplasias o infecciones) dónde pueden encontrarse [1], 2], 8]. Por esta razón, hubiera sido interesante poder haber determinado dentro del grupo DS, qué casos tuvieron un diagnóstico definitivo de SMD, y si alguno de los parámetros de investigación estudiados, hubieran tenido valor en el diagnóstico diferencial. Sin embargo, los elevados tiempos de ejecución de las pruebas complementarias de citometría, biología molecular y citogenética, además de la pérdida de muchos casos por derivación a otros centros para el diagnóstico final, hizo inviable evaluar esta hipótesis. Por tanto, creemos que una vez demostrada la asociación de Neu-GI, IPF y PDW con cambios displásicos, se debería realizar un estudio más ambicioso que incluya el diagnóstico definitivo del paciente, que pueda evaluar el verdadero poder de estos parámetros en el diagnóstico diferencial de SMD.

## Conclusiones

Sólo PDW, IPF y Neu-GI muestran una asociación significativa con la presencia de displasia. Neu-GI presenta el mayor grado de relación, sin que la adición de PDW o IPF aumenten la capacidad predictora significativamente. En pacientes con los criterios del estudio, y con un valor de Neu-GI ≤146ch, la presencia de displasia en sangre periférica resulta altamente problable, haciéndose indispensable la revisión del frotis sanguíneo. Con un valor de Neu-GI >152, la presencia de displasia es altamente improbable.
